# Supplementary material for: The poxvirus F17 protein counteracts mitochondrially orchestrated antiviral responses
Source: Nat Commun. 2023 Nov 30;14:7889. doi: 10.1038/s41467-023-43635-y (PMC10689448; doi:10.1038/s41467-023-43635-y)
Supplement: Supplementary file 1 — Supplementary Information [file 41467_2023_43635_MOESM1_ESM.pdf]

**Supplementary Information For:**

# The poxvirus F17 protein counteracts mitochondrially orchestrated antiviral responses

Nathan Meade[1], Helen K. Toreev[1], Ram Chakrabarty[2], Charles R. Hesser[1], Chorong Park[1], Navdeep S. Chandel[2] and Derek Walsh[1*]

**This File Contains Figures and Legends For:**

Supplementary Figure 1

Supplementary Figure 2

Supplementary Figure 3

Supplementary Figure 4

Supplementary Figure 5

Supplementary Figure 6

Supplementary Figure 7

Supplementary Figure 8

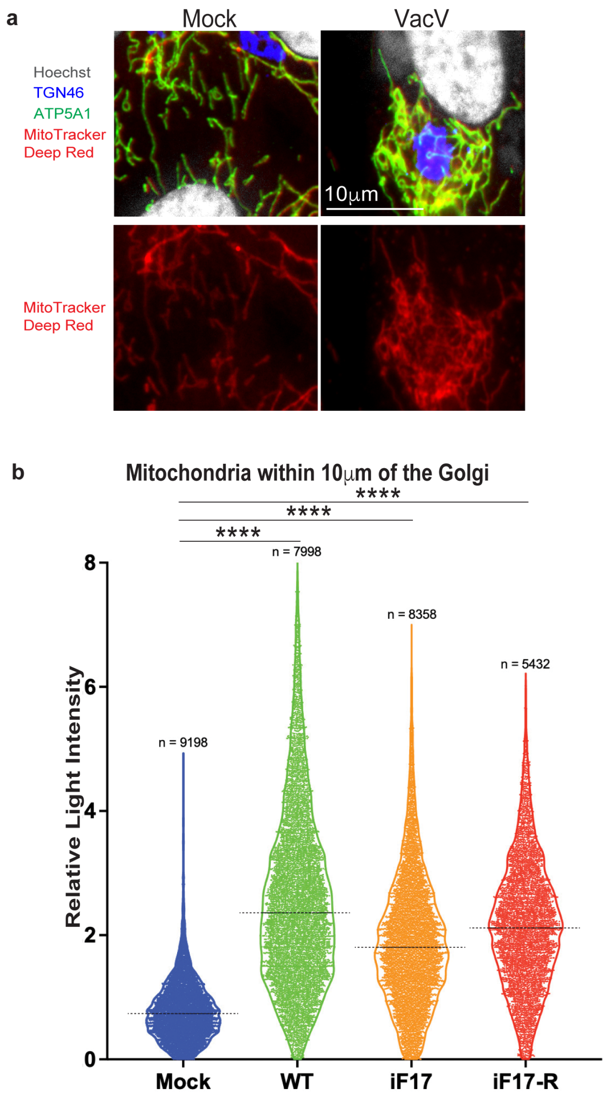


**Supplementary Fig. 1: Mitochondria hyperfuse around the Golgi in poxvirus-infected cells.** NHDFs in **a-b** were infected at MOI 5 for 24h. **a**, Cells were treated with MitoTracker Deep Red prior to fixing and staining for ATP5A1 (green), TGN46 (blue) and Hoechst (gray). Representative merged and MitoTracker images show the usual mixture of elongated and small mitochondria in uninfected cells undergoing normal fusion-fission cycles, versus hyperfused mitochondria that aggregate around the Golgi (stained with TGN46) in infected cells. Note, MitoTracker staining demonstrates sustained MMP in infected cells. **b,** automated slide scan and cell profiler analysis of mitochondrial staining intensity within 10µm of the Golgi. n = as indicated for each group [variable due to random sampling], ****p ≤0.0001, independent two-tailed t-tests. Data are presented as mean values +/- SEM. Source data are provided as a Source Data file.

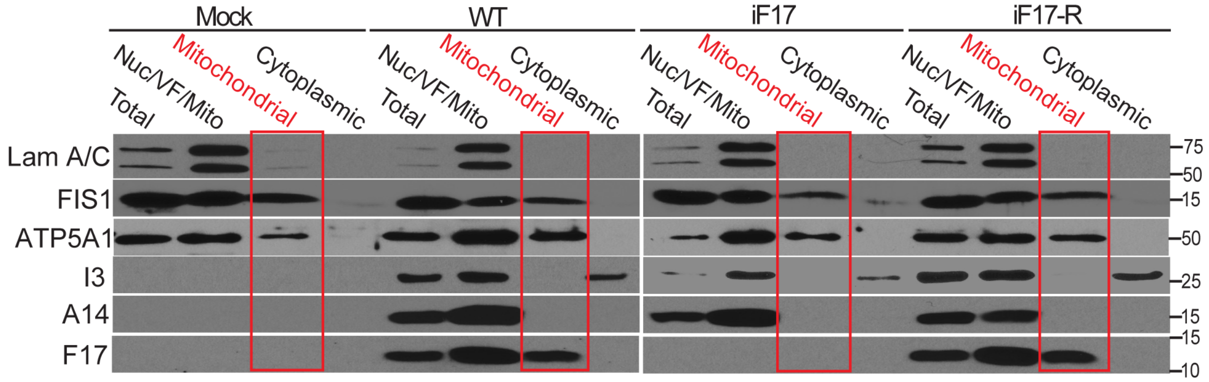


**Supplementary Fig. 2: F17 co-sediments with mitochondria in fractionated cell lysates.** NHDFs were mock infected or infected with the indicated viruses at MOI 5 for 24h. Cell lysates were fractionated and probed for the indicated antigens. Note that nuclei (detected using Lamin A/C) and VFs (detected using structural proteins A14 and F17 itself) co-pellet together with a subpopulation of mitochondria (detected using ATP5A1 and FIS1) that tether to other organelles. However, F17 is also found in the additional, purer mitochondrial fraction while A14 is not. Furthermore, I3, a virus-encoded DNA-binding protein that localizes to VFs and the cytoplasm, was found only in Nuclear/VF/Mito co-sedimentation fractions and cytosolic fractions, but not the purer mitochondrial fraction. Molecular Weight markers are shown in kDa. Data is representative of 3 independent experiments. Source data are provided as a Source Data file.

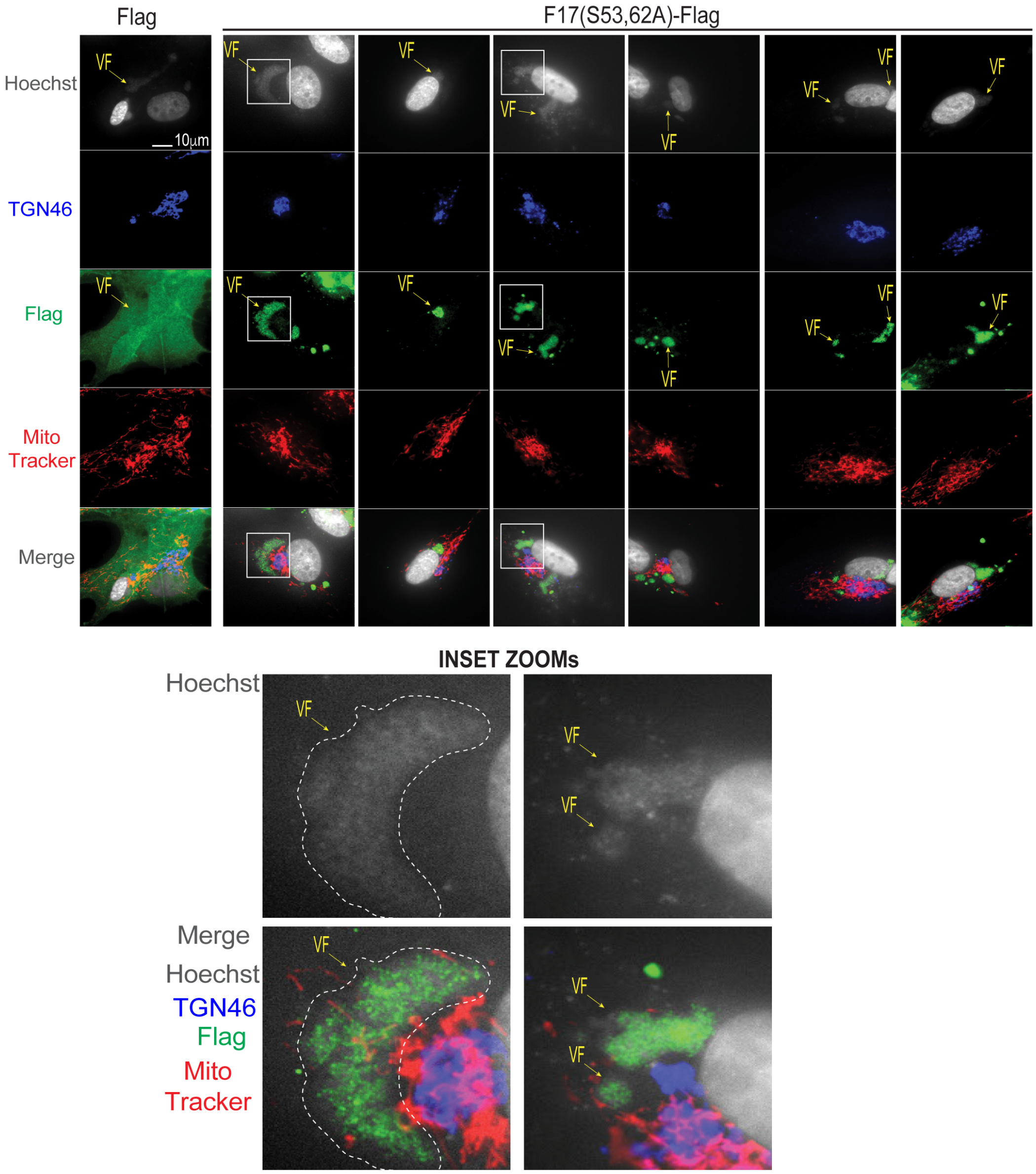


**Supplementary Fig. 3: Non-phosphorylated F17 localizes to Viral Factories.** NHDFs stably expressing Flag control peptide or a Flag-tagged form of F17 that cannot be phosphorylated (S52/63A) were infected with iF17 at MOI 5 for 24h. Cells were incubated with Mitotracker Deep red before being fixed and stained as indicated. Representative images are shown illustrating generalized Flag staining compared with the predominant localization of non-phosphorylated F17 to viral factories; yellow arrows highlight examples of VFs in each case. Inset zooms (lower panels) show the speckled organization of F17 within VFs. Images are representative of phenotypes in >90% of cells in 3 independent experiments.

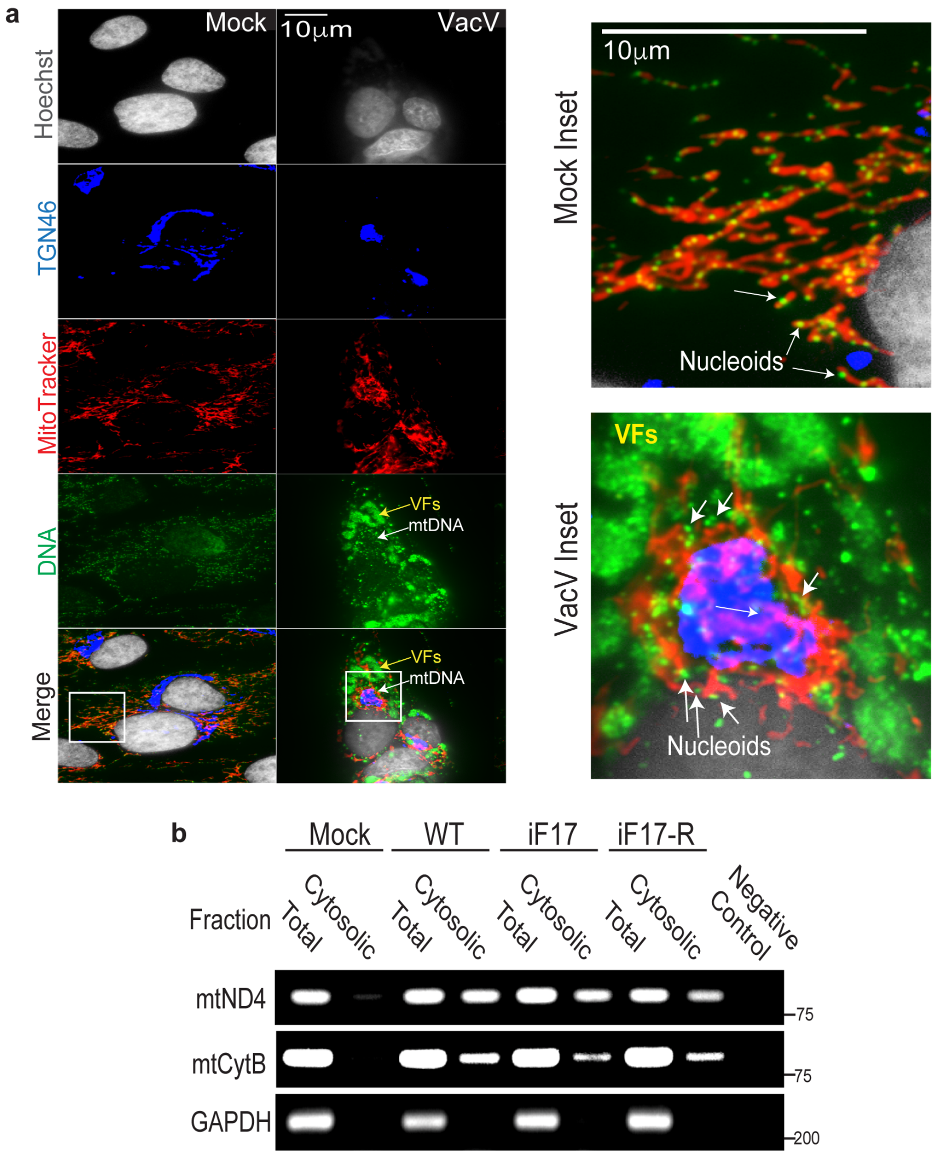


**Supplementary Fig. 4: mtDNA leakage in VacV-infected NHDFs and THP1 monocytes. a,** additional examples of mitochondrial localization and mtDNA release in mock versus VacV-infected NHDFs. Cells were mock infected or infected at MOI 5 and pulsed with MitoTracker Deep Red prior to fixing at 24h.p.i. Samples were co-stained with antibodies against DNA and TGN46, together with Hoechst. Insets show normal mtDNA nucleoids inside mitochondria in uninfected cells while mitochondria around the Golgi of infected cells appear to contain fewer nucleoids that are instead, extra-mitochondrial. Note that the anti-DNA antibody also detects DNA in VFs, labeled in yellow. **b,** THP1 monocytes were mock infected or infected with the indicated viruses at MOI 5 for 30h. PCR analysis of fractionated lysates shows increased mtDNA in the cytosol of cells infected with VacV WT, iF17 or iF17R viruses. Molecular Weight markers are shown in bp. Data in a, b are representative of at least 3 independent experiments. Source data are provided as a Source Data file.

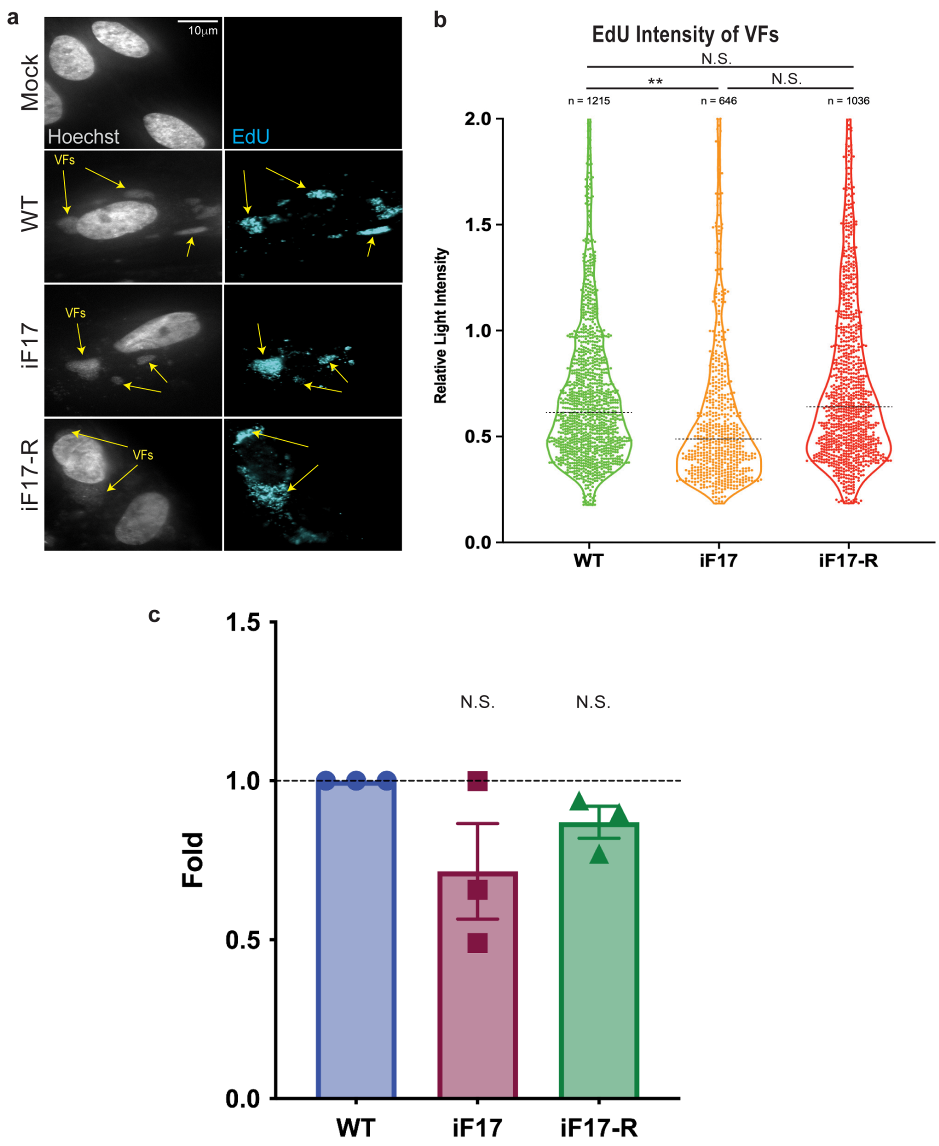


**Supplementary Fig. 5: Viral DNA replication in infected NHDFs. a-c,** NHDFs were infected with the indicated viruses at MOI 5 for 24h. **a-b**, NHDFs were pulsed with Azide-linked EdU prior to fixing. EdU was then detected using CLICK chemistry and cells were further stained with Hoechst and anti-A14 antibody to identify viral factories. **a,** Representative images of EdU-stained viral factories (VFs), with examples highlighted by yellow arrows. **b,** Cell profiler analysis of EdU intensity of individual VFs identified using Hoechst and viral A14 as object identifiers. n = as indicated for each group [variable due to random sampling], ANOVA and Tukey multiple comparison test. Data are presented as mean values +/- SEM. **c,** RT-qPCR analysis of viral DNA levels using the same cytosolic fractions as those used to measure mtDNA in Figure 2a,b, presented as fold relative to WT. Data are presented as mean values +/- SEM. For **b,c**; ns = no significance, **$p \leq 0.01$. Data are representative of 3 or more biological replicates. Source data are provided as a Source Data file.

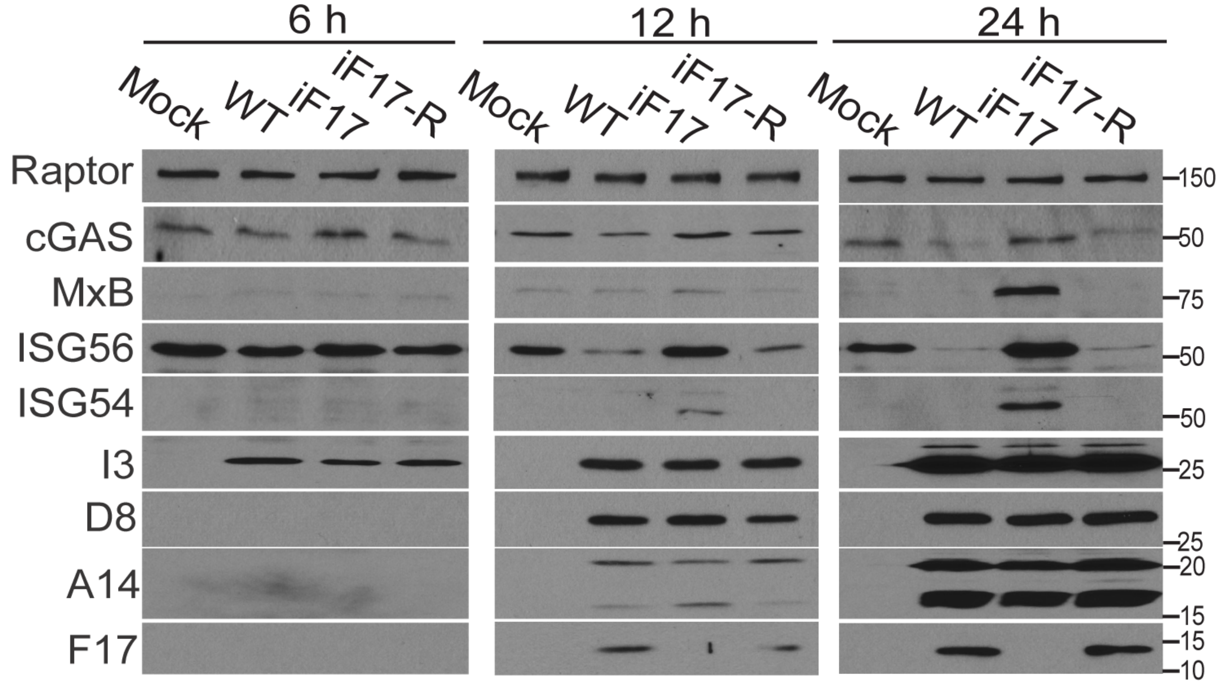


**Supplementary Fig. 6: cGAS destabilization and ISG induction in VacV-infected NHDFs.** NHDFs were mock infected or infected with WT, iF17 or iF17R viruses at MOI 5 for 6h, 12h or 24h. Lysates were analyzed by Western blotting using the indicated antibodies. I3 is an early-intermediate VacV protein that is synthesized throughout the course of infection, while D8, A14 and F17 are late proteins. Quantification of cGAS and ISG levels in replicate experiments is shown in Fig. 2D. Molecular Weight markers are shown in kDa. Source data are provided as a Source Data file.

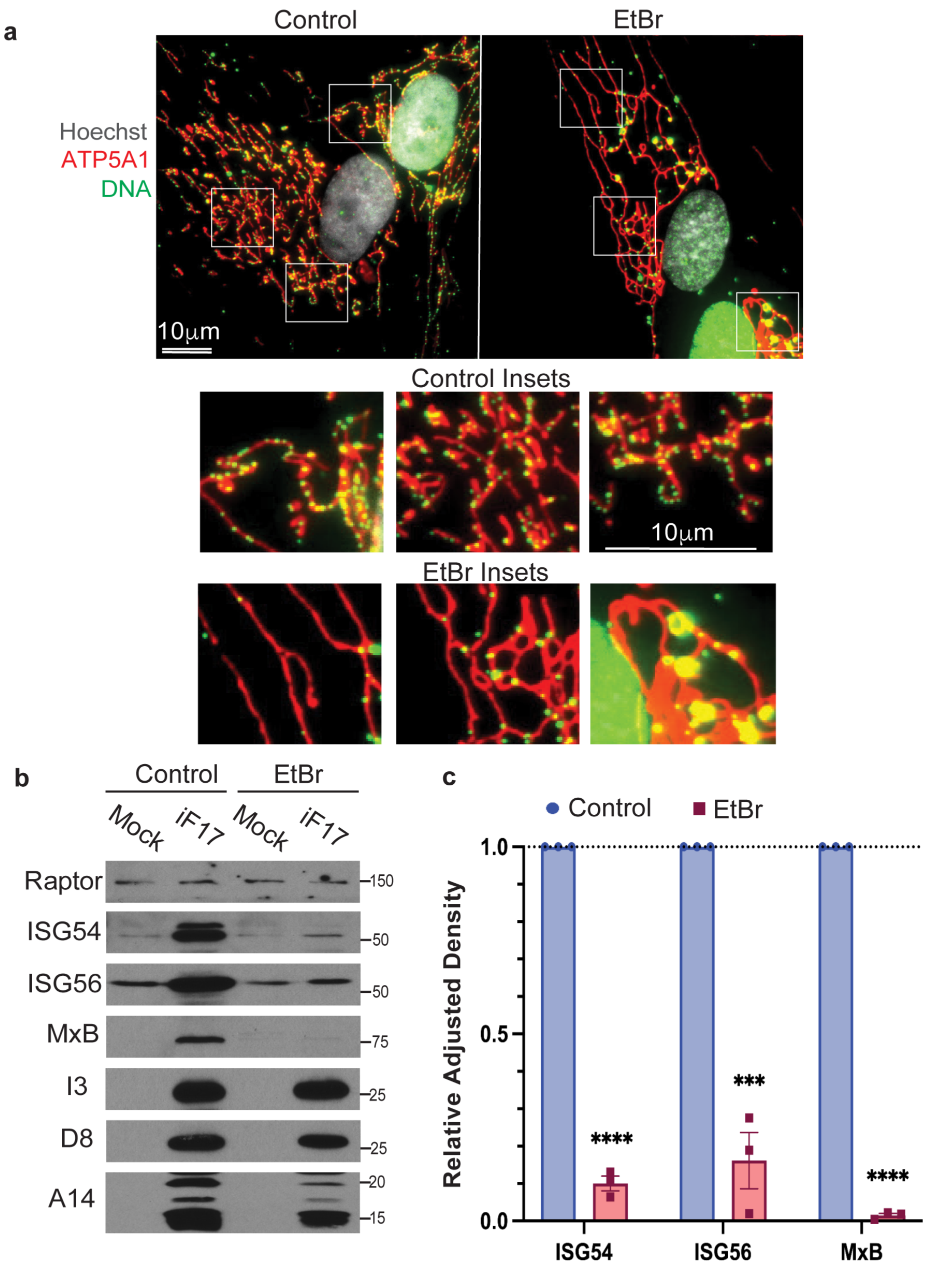


**Supplementary Fig. 7: Treatment with EtBr suppresses ISG responses to iF17 infection.** NHDFs were treated with 1µg/ml EtBr for 48h which was then removed prior to infection at MOI 5 with the indicated viruses. **a,** images of mtDNA nucleoids in control or EtBr-treated NHDFs after 48h treatment prior to infection. zoomed insets show details of various regions within each sample. **b,** representative Western blot analysis of ISG and viral protein expression after infection for 24h. Molecular Weight markers are shown in kDa. **c,** densitometry was used to quantify ISG levels, presented relative to uninfected controls under each condition. n=3 per group, multiple independent t-tests with the corresponding mock for each protein, ***$p \leq 0.001$, ****$p \leq 0.0001$. Data are presented as mean values +/- SEM. Source data are provided as a Source Data file.

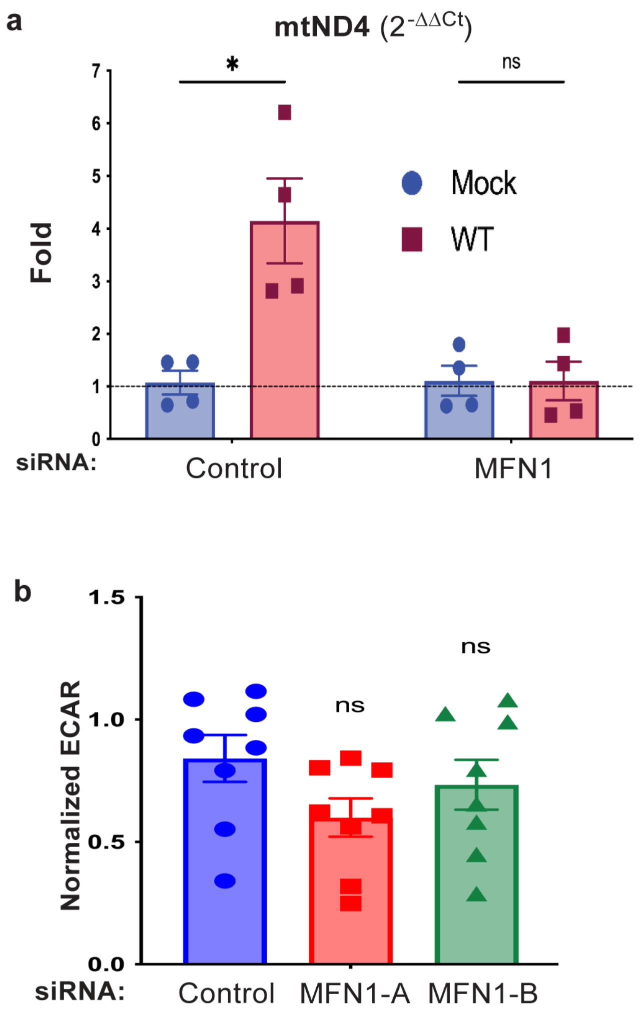


**Supplementary Fig. 8: Effects of MFN1 depletion on mtDNA release and glycolysis.** **a,** NHDFs were treated with control or MFN1 siRNAs prior to mock infection or infection with WT VacV at MOI 5 for 24h. RT-qPCR analysis of fractionated samples showing relative levels of cytosolic mtDNA presented as fold change over control siRNA-treated mock infected cells, arbitrarily set to 1. n = 4 per group, unpaired two-tailed t-test. Data are presented as mean values +/- SEM. **b,** NHDFs were treated with control or MFN1 siRNAs followed by measurement of ECAR. n = 8 per group, independent two-tailed t-tests. Data are presented as mean values +/- SEM. For **a,b**; ns = no significance, *p≤0.05. Source data are provided as a Source Data file.
