# Supplementary material for: The poxvirus F17 protein counteracts mitochondrially orchestrated antiviral responses
Source: Nat Commun. 2023 Nov 30;14:7889. doi: 10.1038/s41467-023-43635-y (PMC10689448; doi:10.1038/s41467-023-43635-y)
Supplement: Supplementary file 3 — Reporting Summary [file 41467_2023_43635_MOESM3_ESM.pdf]

nature portfolio

Corresponding author(s): Derek Walsh

Last updated by author(s): November 7, 2023

Reporting Summary

Nature Portfolio wishes to improve the reproducibility of the work that we publish. This form provides structure for consistency and transparency in reporting. For further information on Nature Portfolio policies, see our Editorial Policies and the Editorial Policy Checklist.

Statistics

For all statistical analyses, confirm that the following items are present in the figure legend, table legend, main text, or Methods section.

n/a	Confirmed	
☐	☒	The exact sample size (*n*) for each experimental group/condition, given as a discrete number and unit of measurement
☐	☒	A statement on whether measurements were taken from distinct samples or whether the same sample was measured repeatedly
☐	☒	The statistical test(s) used AND whether they are one- or two-sided<br>*Only common tests should be described solely by name; describe more complex techniques in the Methods section.*
☒	☐	A description of all covariates tested
☐	☒	A description of any assumptions or corrections, such as tests of normality and adjustment for multiple comparisons
☐	☒	A full description of the statistical parameters including central tendency (e.g. means) or other basic estimates (e.g. regression coefficient) AND variation (e.g. standard deviation) or associated estimates of uncertainty (e.g. confidence intervals)
☐	☒	For null hypothesis testing, the test statistic (e.g. *F*, *t*, *r*) with confidence intervals, effect sizes, degrees of freedom and *P* value noted<br>*Give P values as exact values whenever suitable.*
☒	☐	For Bayesian analysis, information on the choice of priors and Markov chain Monte Carlo settings
☒	☐	For hierarchical and complex designs, identification of the appropriate level for tests and full reporting of outcomes
☐	☒	Estimates of effect sizes (e.g. Cohen's *d*, Pearson's *r*), indicating how they were calculated

Our web collection on statistics for biologists contains articles on many of the points above.

Software and code

Policy information about availability of computer code

Data collection	Imaging was performed using a wide-field Leica DMI6000B-AFC microscope with 100X objective (HC PL APO 100x/1.44NA OIL), X-Cite XLED1 illumination, and an ORCA FLAH 4.0 cMOS camera. Metamorph Microscopy Automation and Image Analysis Software (Molecular Devices) was used for image acquisition and the multi-dimensional acquisition function was used to standardized settings for all images gathered in each independent experiment. For slide scan analysis, an automated stage was used to image the total area of each coverslip. Densitometry measurements were performed on autoradiographs using the Fiji distribution of ImageJ. DNA concentrations were determined using a ThermoFisher Scientific NanoDrop 8000. Lucia luciferase was detected with Quanti-Luc (InvivoGen) on a FluostarOMEGA Plate Reader (BMG LABTECH). Fluorescence readings for ROS and Lactate assays were measured on a FluostarOMEGA Plate Reader (BMG LABTECH). OCR and ECAR assays were performed on Seahorse XF96 PDL Cell Culture Microplates using the Seahorse XFe96 Analyzer.
Data analysis	Microscopy image analysis and processing was done using Metamorph Microscopy Automation and Image Analysis Software (Molecular Devices) and figures were compiled using the Fiji distribution of ImageJ. After collection, slide scan images were then entered into CellProfiler Image Analysis Software using the indicated stains as object identifiers. Mean intensity measurements were used to analyze the data generated and for graphical representation with GraphPad Prism (version 9.0, GraphPad Software Inc.).

For manuscripts utilizing custom algorithms or software that are central to the research but not yet described in published literature, software must be made available to editors and reviewers. We strongly encourage code deposition in a community repository (e.g. GitHub). See the Nature Portfolio guidelines for submitting code & software for further information.

Data

Policy information about availability of data

All manuscripts must include a data availability statement. This statement should provide the following information, where applicable:

- Accession codes, unique identifiers, or web links for publicly available datasets
- A description of any restrictions on data availability
- For clinical datasets or third party data, please ensure that the statement adheres to our policy

The authors declare that the data supporting the findings of this study are available within the article and its Supplementary Information files. Source data are provided with this paper.

Research involving human participants, their data, or biological material

Policy information about studies with human participants or human data. See also policy information about sex, gender (identity/presentation), and sexual orientation and race, ethnicity and racism.

Reporting on sex and gender	N/A
Reporting on race, ethnicity, or other socially relevant groupings	N/A
Population characteristics	N/A
Recruitment	N/A
Ethics oversight	N/A

Note that full information on the approval of the study protocol must also be provided in the manuscript.

Field-specific reporting

Please select the one below that is the best fit for your research. If you are not sure, read the appropriate sections before making your selection.

☒ Life sciences ☐ Behavioural & social sciences ☐ Ecological, evolutionary & environmental sciences

For a reference copy of the document with all sections, see nature.com/documents/nr-reporting-summary-flat.pdf

Life sciences study design

All studies must disclose on these points even when the disclosure is negative.

Sample size	No sample size calculations were performed. Sample size was determined based on commonly used standards for cell biology and sufficient to determine statistical significance. Samples were collected from at least 3 biological replicates. For imaging experiments, the number of cells analyzed was dependent on the number acquired during automated slide scanning and normally involves thousands of cells per sample, performed over at least 3 biological replicate experiments.
Data exclusions	No data exclusion was used.
Replication	Data is representative of at least 3 or more biological replicates, and replicate numbers are indicated for each experiments in their corresponding figure legends.
Randomization	Randomization of fields of view and sample sizes is inherently part of automated image analysis. For all other assays, randomization was not necessary as they involved precise measurements of individual samples.
Blinding	Blinding is inherent to automated image analysis. For all other assays, blinding was not necessary as they involved objective and precise measurements of individual samples.

Reporting for specific materials, systems and methods

We require information from authors about some types of materials, experimental systems and methods used in many studies. Here, indicate whether each material, system or method listed is relevant to your study. If you are not sure if a list item applies to your research, read the appropriate section before selecting a response.

Materials & experimental systems

n/a	Involved in the study
☐	☒ Antibodies
☐	☒ Eukaryotic cell lines
☒	☐ Palaeontology and archaeology
☒	☐ Animals and other organisms
☒	☐ Clinical data
☒	☐ Dual use research of concern
☒	☐ Plants

Methods

n/a	Involved in the study
☒	☐ ChIP-seq
☒	☐ Flow cytometry
☒	☐ MRI-based neuroimaging

Antibodies

Antibodies used

Primary antibodies used in this study were obtained from the following sources:
Rabbit anti-Raptor, Cell Signaling Technology, Cat# 2280
Rabbit anti-cGAS, Cell Signaling Technology, Cat# 15102
Rabbit anti-cGAS, PROTEINTECH, Cat# 26416-1-AP
Rabbit anti-MxA, Cell Signaling Technology, Cat# 37849
Rabbit anti-MxB, PROTEINTECH, Cat# 13278-1-AP
Rabbit anti-MxB, NOVUS BIOLOGICALS, Cat# NBP1-81018
Rabbit anti-ISG56, Cell Signaling Technology, Cat# 14769
Rabbit anti-ISG54, PROTEINTECH, Cat# 12604-1-AP
Sheep anti-TGN46, Bio-Rad, Cat# AHP500
Mouse anti-Flag M2, MilliporeSigma, Cat# F1804
Rabbit anti-Lamin A/C, PROTEINTECH, Cat# 10298-1-AP
Rabbit anti-FIS1, PROTEINTECH, Cat# 10956-1-AP
Rabbit anti-ATP5a1, PROTEINTECH, Cat# 14676-1-AP
Mouse anti-DNA, MilliporeSigma, Cat# CBL186
Rabbit anti-Pink1, Abcam, Cat# ab216144
Rabbit anti-PARK2/Parkin, PROTEINTECH, Cat# 14060-1-AP
Rabbit anti-Mitofusin-1, Cell Signaling Technology, Cat# 14739
Rabbit anti-Mitofusin-2, Cell Signaling Technology, Cat# 9482
Rabbit anti-VDAC1/2, PROTEINTECH, Cat# 10866-1-AP
Mouse anti-β-Actin, Cell Signaling Technology, Cat# 3700
Rabbit anti-CytB, PROTEINTECH, Cat# 55090-1-AP
Rabbit anti-TFAM, PROTEINTECH, Cat# 22586-1-AP
Rabbit anti-PARP, Cell Signaling Technology, Cat# 9542
Rabbit anti-Cleaved Caspase-3 (Asp175), Cell Signaling Technology, Cat #9661
Rabbit anti-VacV F17 was raised against full length purified F17 protein by ABclonal, USA
Mouse anti-VacV I3 was a kind gift of Dr. David Evans
Mouse anti-VacV E3 was a kind gift of Dr. Jingxin Cao
Mouse anti-VacV D8 was a kind gift of Dr Paula Traktman
Mouse anti-VacV A14 was a kind gift of Dr. Yan Xiang

Secondary antibodies used in this study were obtained from the following sources;
Anti-Mouse IgG, HRP, Millipore Sigma, Cat# NA931V
Anti-Rabbit IgG, HRP, Millipore Sigma, Cat# NA934V
Donkey Anti-Mouse secondary Alexa 488, Thermo Fisher Cat# A21202
Donkey Anti-Mouse secondary Alexa 555, Thermo Fisher Cat# A31570
Donkey Anti-Mouse secondary Alexa 647, Thermo Fisher Cat# A31571
Donkey Anti-Rabbit secondary Alexa 488, Thermo Fisher Cat# A21206
Donkey Anti-Rabbit secondary Alexa 555, Thermo Fisher Cat# A31572
Donkey Anti-Rabbit secondary Alexa 647, Thermo Fisher Cat# A31573
Donkey Anti-Sheep secondary Alexa 488, Thermo Fisher Cat# A11015
Donkey Anti-Sheep secondary Alexa 555, Thermo Fisher Cat# A21436

Validation

Vendor validation information is available online for the following antibodies:
Rabbit anti-Raptor, Cell Signaling Technology, Cat# 2280
Rabbit anti-cGAS, Cell Signaling Technology, Cat# 15102
Rabbit anti-cGAS, PROTEINTECH, Cat# 26416-1-AP
Rabbit anti-MxA, Cell Signaling Technology, Cat# 37849
Rabbit anti-MxB, PROTEINTECH, Cat# 13278-1-AP
Rabbit anti-MxB, NOVUS BIOLOGICALS, Cat# NBP1-81018
Rabbit anti-ISG56, Cell Signaling Technology, Cat# 14769
Rabbit anti-ISG54, PROTEINTECH, Cat# 12604-1-AP
Sheep anti-TGN46, Bio-Rad, Cat# AHP500
Mouse anti-Flag M2, MilliporeSigma, Cat# F1804
Rabbit anti-Lamin A/C, PROTEINTECH, Cat# 10298-1-AP
Rabbit anti-FIS1, PROTEINTECH, Cat# 10956-1-AP
Rabbit anti-ATP5a1, PROTEINTECH, Cat# 14676-1-AP
Mouse anti-DNA, MilliporeSigma, Cat# CBL186

Rabbit anti-Pink1, Abcam, Cat# ab216144
Rabbit anti-PARK2/Parkin, PROTEINTECH, Cat# 14060-1-AP
Rabbit anti-Mitofusin-1, Cell Signaling Technology, Cat# 14739
Rabbit anti-Mitofusin-2, Cell Signaling Technology, Cat# 9482
Rabbit anti-VDAC1/2, PROTEINTECH, Cat# 10866-1-AP
Mouse anti-β-Actin, Cell Signaling Technology, Cat# 3700
Rabbit anti-CytB, PROTEINTECH, Cat# 55090-1-AP
Rabbit anti-TFAM, PROTEINTECH, Cat# 22586-1-AP
Rabbit anti-PARP, Cell Signaling Technology, Cat# 9542
Rabbit anti-Cleaved Caspase-3 (Asp175), Cell Signaling Technology, Cat #9661
Anti-Mouse IgG, HRP, Millipore Sigma, Cat# NA931V
Anti-Rabbit IgG, HRP, Millipore Sigma, Cat# NA934V
Donkey Anti-Mouse secondary Alexa 488, Thermo Fisher Cat# A21202
Donkey Anti-Mouse secondary Alexa 555, Thermo Fisher Cat# A31570
Donkey Anti-Mouse secondary Alexa 647, Thermo Fisher Cat# A31571
Donkey Anti-Rabbit secondary Alexa 488, Thermo Fisher Cat# A21206
Donkey Anti-Rabbit secondary Alexa 555, Thermo Fisher Cat# A31572
Donkey Anti-Rabbit secondary Alexa 647, Thermo Fisher Cat# A31573
Donkey Anti-Sheep secondary Alexa 488, Thermo Fisher Cat# A11015
Donkey Anti-Sheep secondary Alexa 555, Thermo Fisher Cat# A21436

Additional validation of antibodies is shown in this manuscript as follows:
Validation of MFN1 and cGAS antibodies was performed using siRNA- or CRISPR-mediated mRNA/gene depletion.
Specificity of PARP-1 and Capase-3 antibodies was further validated by inducing apoptosis in cells using cycloheximide.
Staining specificity of golgi, nuclear and mitochondrial antibodies were validated by their expected localization and cellular fractionation patterns.
Validation of antibodies against ISGs was confirmed by their induction upon iF17 infection and by failure of their induction in cGAS Knockout cells. Further validation of ISG antibody specificity using IFN treatment was shown previously in Meade et al, Cell, 2018.
In the case of antibodies against viral proteins, namely F17 antibody that was generated in-house and gifted antibodies against E3, I3, D8 and A14, specificity was validated through the absence of antigen signal in uninfected cells.

Eukaryotic cell lines

Policy information about cell lines and Sex and Gender in Research

Cell line source(s)	Primary Normal Human Dermal Fibroblasts (NHDFs) isolated from male neonatal foreskin were purchased from Lonza (Cat# CC-2509). Male-derived THP1 Dual reporter cells were obtained from Invivogen (Cat# thpd-nfis; Cat# thpd-kocgas). Female-derived HEK293T Cells were obtained from the ATCC. Female African green monkey BSC-40 cells were a gift of Dr. Ian Mohr, NYU School of Medicine, New York, USA.
Authentication	All cell types used for primary studies were authenticated by the manufacturer. Cell types used to generate viral stocks, namely HEK293A cells or BSC-40 cells were validated by the original supplier. Tissue culture practices are used that prevent cross-contamination of cell lines and no further authentication was performed in-house (although routine genomic and proteomic experiments align with their original authentication). No commonly misidentified cell lines were used in this study.
Mycoplasma contamination	All cell cultures were routinely screened and verified to be free of mycoplasma using DNA staining and imaging, as well as regular testing using commercial mycoplasma test kits.
Commonly misidentified lines (See ICLAC register)	N/A
